# Editorial: Negative Regulators of Innate Immunity and Their Role in Host Responses to Injury and Infection

**DOI:** 10.3389/fimmu.2022.891919

**Published:** 2022-03-28

**Authors:** Shrikant R. Mulay, Maciej Lech

**Affiliations:** ^1^ Pharmacology Division, Council of Scientific and Industrial Research (CSIR)-Central Drug Research Institute, Lucknow, India; ^2^ LMU Klinikum, Medizinische Klinik und Poliklinik IV, Ludwig-Maximilians-Universität Munich, München, Germany

**Keywords:** homeostasis, allostasis, inflammation, infection, injury

## Introduction

Every cell type can sense danger and at the same time display a plethora of different mechanisms that allow it to maintain or restore homeostasis. It is not surprising that immune cells that in their function per se are sensitive to danger signals play a predominant role in keeping the immune balance. Cells use various extra- and intracellular factors to regulate inflammation making it a central component of the system that orchestrates the immune responses since a tightly regulated immune system provides stability through constancy. Efficient and adequate regulation of immune responses requires various modulatory mechanisms that trigger allostasis, defined as the ability to accomplish stability through change. Defective immune responses frequently lead to severe (1) autoinflammatory or (2) autoimmune reactions that may both be systemic or tissue-specific. Autoinflammatory disorders are characterized by self-directed inflammatory responses and acute inflammation as a consequence of the unregulated innate immune system. Autoimmune diseases result from the loss of immune tolerance against self-antigens. They are characterized by the existence of autoreactive T and B cells and are often described as triggered by the impairment of adaptive immunity alone. In both cases, a lack of negative regulation of immune responses might be responsible for the onset of inflammatory disease. Conserved regulatory mechanisms modulate the immune response and bridge the gap between innate and adaptive immunity as they can terminate first inflammatory responses and initiate subsequent development of adaptive immunity. The uncontrolled influx and activation of the immune cells associated with the production of pro-inflammatory cytokines might, in consequence, lead to a prolonged presentation of intracellular antigens, and the development of autoantibodies against particular cell compartments are produced are often the cause of severe diseases. Despite a significant number of experimental studies the complex mechanisms of immune dysregulation are not well understood. This Frontiers Research Topic represents a collection of articles that focused on innate and adaptive mechanisms that contribute to local homeostasis and tissue-specific responses to the infection, injury, and inflammation.

## Homeostasis, Allostasis, and the Key Role of Negative Regulators of Inflammation

Wang and Liu reviewed the role of a key molecule in the complement system C4 in infections and autoimmune diseases. The authors gave a comprehensive overview of the regulation of complement C4 activation and presented the immunomodulatory role of C4 linking microbial infections and autoimmune disorders. Supino et al. focused on the negative regulation of the IL-1 system. The IL-1 family of cytokines and receptors is associated with a broad spectrum of immunological and inflammatory responses. They discussed the hallmarks of two key regulatory receptors of the IL-1 system, IL-1R2, the first decoy receptor identified, and IL-1R8, a pleiotropic regulator of different IL-1 family members and co-receptor for IL-37, the anti-inflammatory member of the IL-1 family.

Nucleotide‐binding oligomerization domain (NOD)‐like receptors (NLRs) are a specialized group of cytosolic pattern recognition receptors (PRRs) that represent a crucial component of the host innate immune system. They include NLRA, NLRB, NLRC, NLRP, and NLRX superfamilies and are responsible for monitoring the intracellular microenvironment, mediating inflammation, and pathogen clearance (1). Quenum et al. demonstrated the importance of nucleotide-binding leucine-rich repeat-containing receptor (NLR) family protein-5 (NLRC5) in liver homeostasis. The authors proved that NLRC5 regulated hepatic inflammatory response upon injury but did not affect liver fibrosis. Moreover, NLRC5-deficient livers showed increased phosphorylation of the NF-κB subunit p65 increased expression of F4/80 (Adgre1), a marker of tissue-resident macrophages. Further supporting the role of NLRs in homeostasis and inflammation, Kastelberg et al. focused on Nlrx1 as a critical regulator of immune signaling in pulmonary fungal infection using the clinically relevant fungus Aspergillus fumigatus. They showed that loss of Nlrx1 resulted in a decreased ability of host cells to process A. fumigatus conidia in a cell type-specific manner and limited ability to generate superoxide and/or generic reactive oxygen species during specific responses to fungal PAMPs. They proved that during fungal pulmonary infections Nlrx1 is responsible for the regulation of cell metabolism by affecting the glycolysis process. Besides NLRs, innate immune cells are equipped with a set of other cytosolic sensors so-called inflammasomes, which are responsible for the detection of pathogens and danger signals. Chowdhury et al. dedicated their experimental undertake to a mechanism that restricts non-canonical inflammasome activation. They elegantly demonstrated that the caspase-11 inflammasome in mouse and human macrophages (Mϕ) is negatively controlled by the zinc (Zn2+) regulating protein, metallothionein 3 (MT3). MT3 enhanced intracellular Zn2+ resulting in inhibition of the TRIF-IRF3-STAT1 pathway and restricting of caspase-11 effector function. Other regulatory mechanisms involved in controlling inflammation and immunity to pathogens refer to immune responses against viruses and parasites. Peng et al. identified pleckstrin homology-like domain, family A, member 1 (PHLDA1) as a negative regulator of LPS-induced proinflammatory response. This study evidenced that PHLDA1 suppresses TLR4/MyD88 signaling pathway using the interaction with Toll Interacting Protein (TOLLIP).

Lee et al. investigated the expression of ephrinA1/ephA2 in normal mucosa and inflamed sinonasal mucosa of chronic rhinosinusitis (CRS) patients. The authors described a novel role of ephrinA1/ephA2 signaling in antiviral innate immune response in the sinonasal epithelium. They proved that rhinovirus (RV) infection or poly (I:C) treatment induced chemokine secretion which was attenuated by ephA2 inhibitor. The production of antiviral mediators including type I and type III IFNs enhanced upon blocking ephA2 suggesting its role as a negative regulator of antiviral immunity. Kalia et al. considered an alternative approach to highlight the importance of immune modulation in fighting the disease. The authors investigated the protein PbTIP from Plasmodium berghei. PbTIP is a homolog of the human T cell immunomodulatory protein (TIP) that suppresses host immune responses. The shed PbTIP is a perfect example of a molecular mimic that modulates macrophage responses during the parasitic infection increasing the survival of the parasite.

Feng et al. investigated the mechanism by which neutrophil extracellular traps (NETs) affect spinal cord injury (SCI). NETs regulation is critical for neutrophils to exert their immunological activity. They showed that neutrophils promote neuroinflammation, whereas successful restriction of inflammation ease secondary damage, thus hindering scar formation and improving recovery after SCI. Both inhibitions of NETs formation by peptidylarginine deiminase 4 (PAD4) inhibitor and disruption of NETs by DNase 1 showed positive effects.

## Targeting Inflammation to Regain Homeostasis

Further articles in this Research Topic presented the anti-inflammatory approach as target for pharmacological intervention. Rosa-Guerrero et al. described the successful response to high-dose intravenous immunoglobulin (IVIG) combined with steroid pulses in the case of Covid-19 pneumonia in a single-kidney transplanted patient. The authors described the clinical efficacy and anti-inflammatory and immunosuppressive potential of steroids and contributed to the special issue with an interesting example of treatment of inflammatory disease.

Complementary to the above-mentioned work, Rossi et al. illustrate an example of new phenotypic abnormality in four consanguineous patients with a mutation in affecting TNFAIP3 (A20), a central negative regulator of NF-κB signaling pathway. They identified a novel heterozygous frameshift mutation (p.His577Alafs*95) that introduced a premature stop codon in the zinc finger domain of A20/TNFAIP3, leading to putative haploinsufficiency of the protein. The authors linked the mutation to a predominantly autoimmune phenotype with recurrent fever episodes. The mutation leads to decreased levels of A20 in blood cells and higher levels of NF-kB phosphorylation, as well as increased production of the proinflammatory cytokines IL-1β, IL-6, TNF-α, and hyperactivation of the IFNγ signaling pathway.

## Summary and Perspective

Various studies challenge the role of homeostasis and mechanisms of immune control in health and disease, as well as their substantial potential as a target in clinical applications. Allostatic load and loss of immune homeostasis might aggravate the tissue damage and affect the tissue repair during tissue injury. A better understanding of molecules that modulate immune processes could lead to the development of innovative therapies in the future.

## Author Contributions

SM and ML wrote the article. Both authors contributed to the article and approved the submitted version.

## Conflict of Interest

SM is now an employee for AstraZeneca (Biopharmaceuticals R&D, Cambridge, UK).

The remaining authors declare that the research was conducted in the absence of any commercial or financial relationships that could be construed as a potential conflict of interest.

## Publisher’s Note

All claims expressed in this article are solely those of the authors and do not necessarily represent those of their affiliated organizations, or those of the publisher, the editors and the reviewers. Any product that may be evaluated in this article, or claim that may be made by its manufacturer, is not guaranteed or endorsed by the publisher.

## References

[1] Chen G Shaw MH Kim YG Nunez G. NOD-Like Receptors: Role in Innate Immunity and Inflammatory Disease. Annu Rev Pathol (2009) 4:365–98. doi: 10.1146/annurev.pathol.4.110807.092239 18928408

